# Time for a Re-Think? The Rationale for Multi-Component Intervention to Prevent Malnutrition in At-Risk Community-Dwelling Older Adults

**DOI:** 10.3390/geriatrics9050124

**Published:** 2024-09-23

**Authors:** Johnny Naylor, Alexandra M. Johnstone, Phyo K. Myint

**Affiliations:** 1 Institute of Education in Healthcare and Medical Sciences, School of Medicine, Medical Sciences and Nutrition, University of Aberdeen, Scotland AB25 2ZN, UK; johnny.naylor1@nhs.scot; 2 The Rowett Institute of Nutrition and Health, University of Aberdeen, Scotland AB25 2ZD, UK; alex.johnstone@abdn.ac.uk; 3 Institute of Applied Health Sciences, School of Medicine, Medical Sciences and Nutrition, University of Aberdeen, Scotland AB25 2ZN, UK

Dietary strategies for early intervention in older adults are highly desirable, as they encourage individuals to retain a good functional status despite morbidity. There are strong associations between a poor nutritional status and functional impairment [1]. The Randomised Control Trial by Muangpaisan et al. adds to a body of evidence reporting the benefits of Oral Nutritional Supplements (ONS) in an ageing population [2]. The benefits of ONS in patients within the hospital environment and in residential care have been reported [3]. In their paper, Muangpaisan et al. importantly focus on community-dwelling older adults at risk of malnutrition, thereby providing new evidence regarding the use of early intervention with ONS in the Thai population.

Malnutrition is usually under-recognised, despite evidence suggesting that it is a potentially preventable condition. Globally, it is estimated that 26.5% of older adults (>60 years) in the community setting are at risk of malnutrition [4]. The Thai population are somewhat overrepresented, with 54.8% of community-dwelling older adults found to be at risk [5]. This issue is compounded by the growth of its ageing population [6], a trend which can also be identified globally.

The increase in anorexia observed in older patients is well-recognised, with a plethora of evidence reporting increased mortality and an impaired immune status in malnourished individuals [7]. The optimisation of nutrition and hydration in older adults demands the implementation of multiple strategies across a variety of settings. This is often an invisible challenge in the care home environment [8]. The negative impact of these spaces on individuals’ quality of life (QoL) should not be understated, with high rates of functional impairment and disability related to malnutrition [1]. ONS could aid in the supplementation of individuals’ regular food intake and improve their nutritional status. However, an increase in macronutrient intake may not be enough in isolation. Enhancing health literacy through dietary counselling (DC) for older adults and their support network is another key strategy recommended by current guidance [9]. How regional policy, food security and private entities within the industry can impact older adults positively and negatively must also be considered.

The outcomes of nutritional studies often focus on weight gain, the Malnutrition Universal Screening Tool (MUST) or dietary diversity scores. Each can be an important aspect nutrition; however, they are not directly patient-centred and fall short of representing either a reduction in mortality or an improvement in QoL.

Muangpaisan et al. conducted a prospective, open-label, randomised controlled study across two centres in Thailand over 60 days [2]. The intervention group, comprising 194 older adults, received ONS twice daily, with DC at days 0 and 30. The second group received only DC over the same time interval. Due to the exclusion criteria used in this study, most participants (*n* = 182) had a Charlson Comorbidity Score of 0 (indicating low comorbidity or chronic disease) and most participants were female (*n* = 161). A large and significant difference in the change in body weight between the two groups was reported, both on day 30 and day 60, with a relative increase of 0.80 ± 01.9 kg (*p* < 0.0001) and 1.50 ± 0.22 kg (*p* < 0.0001), respectively; this was in favour of intervention with ONS, with DC vs. DC alone [2]. The control group, receiving only DC, also reported a significant increase in body weight by the end of the study period (0.16 ± 0.16 kg at day 60). These results agree with a previous systematic review of similar studies that reported an increase in weight gain with ONS in the intervention group [10]. Muangpaisan et al. also report a reduction in MUST risk to 0 by day 60 in 19.8% of the intervention group and 6.5% of the control group. This is likely reflective of the overall weight gain of participants caused by an increase in the intake of macronutrients due to ONS. This shows that patient compliance with the intervention was excellent for both arms of the study. Other reported benefits such as dietary diversity scores were not examined in the outcomes of the study, as both groups were given DC. However, the benefits of this education should not be understated in comparison to absolute weight gain. A study conducted in Sri Lanka demonstrated the potential short-term improvement in dietary diversity facilitated by nutritional education [11].

A Cochrane review of ONS with or without nutritional education in adults cited that most outcomes had low-certainty evidence [10]. The major limitations of current research on ONS as an intervention have been the small sample sizes used and the short intervention duration [10]. The potential for reporting bias was also high, as many studies were open label. Successful outcomes have centred on weight gain or other similar measures of nutritional status. Overall healthcare outcomes such as hospitalisation and mortality, or patient-centred outcomes such as functional status, lack sufficient data to show statistically significant results [10].

Weight gain is not a patient-centred outcome used to determine nutritional success in older adults, especially without an assessment of body composition. Sarcopenia (loss of muscle mass) is a key area for which increasing individuals’ protein intake, either through ONS or DC, could be related to improvements in functional status [12]. Muscle mass could be included as an outcome in more nutritional studies. Special attention should also be paid, perhaps in parallel arms when designing nutritional studies, to older adults living with significant vascular risk factors, dementia, depression, diabetes and neurodegenerative conditions. These groups, whilst more challenging to include, are most at risk of malnutrition due to their physical and social vulnerability. The European Society for Clinical Nutrition and Metabolism (ESPEN) currently recommends that those at risk of malnutrition should be offered ONS only when DC and food fortification are insufficient [9]. However, the priorities of the individual in the recommendation of ONS should be considered to support a holistic approach to care.

In conclusion, future efforts to examine ONS in older adults should include greater numbers of comorbid individuals and measures of functional status and body composition, alongside current outcomes and advances in size and length. Further, researchers should also consider how their outcomes could be used to change not only current guidance, but regional policy and industry. Food security already has a greater impact on nutrition than any other factor globally, and this impact is only likely to increase as the global effects of climate change develop [13]. Evidence-based, person-centred and best practice approaches that recognise how and when to best support older adults through these challenges are vital to prevent malnutrition.
